# Supplementary material for: Wild jackdaws can selectively adjust their social associations while preserving valuable long-term relationships
Source: Nat Commun. 2023 Sep 11;14:5103. doi: 10.1038/s41467-023-40808-7 (PMC10495349; doi:10.1038/s41467-023-40808-7)
Supplement: Supplementary file 1 — Supplementary Information [file 41467_2023_40808_MOESM1_ESM.pdf]

Wild jackdaws can selectively adjust their social associations while preserving valuable long-term relationships

Michael Kings [1]*, Josh J. Arbon [1, 2]*, Guillam E. McIvor [1], Martin Whitaker [3], Andrew N. Radford [2], Jürgen Lerner [4, 5] & Alex Thornton [1]*

[1] Centre for Ecology and Conservation, University of Exeter, Penryn Campus, Treliever Road, Penryn, TR10 9FE, UK

[2] School of Biological Sciences, University of Bristol, 24 Tyndall Avenue, Bristol, BS8 1TQ, UK

[3] technologywithin, Chevron Business Park, Limekiln Lane, Holbury, Southampton, SO45 2QL, UK

[4] Department of Computer and Information Science, University of Konstanz, 78457, Konstanz, Germany

[5] HumTec Institute, RWTH Aachen University, 52062, Aachen, Germany

*Correspondence to: mkings1024@gmail.com; josh.arbon@bristol.ac.uk; alex.thornton@exeter.ac.uk

Supplementary Information

Contents

Supplementary Methods (pp. 3 - 7)

1. RFID data processing (pp. 3 - 4)
2. REM specifications (pp. 4 - 6)
3. Permutation procedure validation (p. 6)
4. Visualization of REM output (pp. 6 - 7)

Supplementary Notes (pp. 8 - 10)

1. Food preference test (p. 8)
2. Distribution of individuals' task activity during experimental days (p. 8)
3. Individual and dyad properties and treatment distributions (pp. 8 - 9)
4. Expected triad composition in experimental networks (p. 9)
5. Triadic closure statistics in Eventnet 0.5.2 (p. 9)
6. Limitations of permutation procedures based on constrained label swaps (p. 10)

Supplementary Figures (pp. 11 – 16)

1. Distribution of overall task experience amongst individuals and dyads (p. 11)
2. Outline of permutation procedure (p. 12)
3. Data-stream permutation variants applicable to REM datasets (p. 13)
4. Expected class composition of triads in experimental networks (p. 14)
5. Permutation procedure diagnostics (p. 15)
6. Drawbacks of permuting datasets using serial pairwise label-swaps (p. 16)

Supplementary Tables (pp. 17 - 28)

A. REM output tables (Supplementary Tables 1 – 8; pp. 17 – 24)
B. Supplementary Movie transcript (Supplementary Table 9; pp. 25 - 26)
C. Glossary of common REM terms (Supplementary Table 10; p. 27)
D. Event concatenation (Supplementary Table 11; p. 28)

Supplementary Methods

1. RFID data processing

Prior to data analysis, data processing was performed to improve the accuracy of estimates of event durations and arrival latencies, where arrival latency was defined as the difference in time between the arrival of the first individual of a dyad and the onset of a social association event (as triggered by the arrival of the second member of the dyad). Due to occasional mis-alignment of PIT-tags with the RFID antennae on the feeding perches, some event durations were likely to be underestimated. Incorporation of a smoothing function in the 'Darwin Board' code, which caused a departure from a feeder to only be registered if the individual was immediately replaced by another bird or if more than two seconds had elapsed since the individual's PIT-tag was detected on the perch, reduced but did not remove these errors in estimates of event duration. Interpolation was therefore necessary to improve duration estimates such that questions concerning change in dyadic coordination at the task could be addressed.

Following data collection, we applied a rule to concatenate events in the dataset under certain circumstances to provide the interpolation needed to correct for the error in visit duration estimates. If two events by the same entity (individual or dyad) were spaced by less than 15 seconds, with no intervening event by another entity, then events were concatenated into a single event with a start time given by the onset of the first event and an end time given by the offset of the second event. A similar concatenation function was also utilized for estimation of arrival latencies. The 15 second rule was applied to the arrival of the first individual, such that if that individual had an event on the feeder within 15 seconds of its most recent event before the onset of the association event, then the arrival latency was instead calculated as the difference in time between the onset of the earlier event and the onset of the association event. A 15 second window was chosen for concatenation as it was the functional resolution of the task (the minimum duration of a non-default task state (the HIGH reward state) was 15 seconds), meaning that repeated events could not alter the state of the task within a 15 second window following the onset of an event, provided the identities of the participants did not change. Resulting estimates of event duration and arrival latency were discrete counts rounded down to the nearest second (e.g., 0 = 0-1, 1 = 1-2, 2 = 2-3 seconds etc).

Concatenation had a significant effect on the balance of the number and duration of successful and unsuccessful association events. Following concatenation, the proportion of association events that were successful was significantly lower than for the dataset without interpolation applied (Supplementary Table 11; McNemar's Chi-squared test with continuity correction, $X^2 = 370$, $p<0.001$). In contrast, the proportion of the total duration of all events (in seconds) that consisted of time spent engaging in successful events significantly increased following concatenation (Supplementary Table 11; McNemar's test, $X^2 = 1269$, $p<0.001$). Though concatenation had a significant effect on the balance of successful and unsuccessful association events in the dataset, it was deemed sufficiently conservative as to be an acceptable procedure because it decreased, rather than increased, the proportion of successful social coordination events in the dataset (Supplementary Table 11).

Processes

1. Missing departure labels needed to be fixed due to a missed 'write to LOG file' function call in the 'Darwin Board' C code. Departures were labelled when tag ID changed from present ID to blank, but not when the change went from present ID to another ID without a transition via a blank. Therefore, whenever replacement occurred within approx. 2 seconds (buffer threshold of

D.B.), the departure label was missing. These missing DEPARTURE labels were replaced during processing in R.

2. Interpolation was applied to improve estimates of visit duration and arrival latency (explanation above).
3. Events that contained individuals that did have a PIT-tag, but did not yet have a treatment class assigned (and so were unable to affect task state) at the time the event was recorded were filtered out of the dataset. These consisted of events engaged in by an individual on the day that it was ringed/re-ringed (i.e., before the task was updated with new RFID codes).
4. 37 events were removed from the dataset for which the event type label (successful association event or failed association event) did not match the treatment class assignments of the participants (due to duplication of RFID codes and/or human error when noting class assignment). 19 of these events were failed association events and 18 were successful.

2. REM specifications

Models 1 & 2: Relationship of individual task performance with: an individual's number of repeated events with same-class and different-class participants (model 1), and the number of same-class and different-class partners with which an individual had associated on multiple occasions (model 2).

Two REMs were used to investigate the role of partner-discrimination in individual task performance. To perform well at the task, individuals needed to learn to avoid associating with participants with which they had previously been unsuccessful (different-class individuals) and associate with greater frequency with those that they had been successful with (same-class individuals). The models therefore addressed the frequency with which individuals engaged in repeated associations with familiar (at least one previous event) same-class and different-class task partners. Model 1 addressed the relationship between an individual's prior history of successful and unsuccessful association with familiar task partners, as represented by overall quantity of each type of event, and future success (Supplementary Table 1). Model 2 was used to investigate the relationship between future performance and the distribution of same-class and different-class partners with which an individual had associated on multiple occasions (Supplementary Table 2). This model contained counts of the numbers of same-class and different-class participants with which an individual had associated multiple times. For both models, incidence rate ratios represented the likelihood of an individual being observed engaging in successful association events in the future (relative to the baseline rate). In addition, this likelihood of future success was conditional upon the individual in question being the second member of a pair to arrive at a feeder, so triggering the onset of an association event (see 'Conditioned on Source/Target' in Supplementary Table 10).

Model 3: Changes in dyadic association rates over the course of the experiment (for different dyad types: same/different treatment class).

This REM was fitted to examine the change in the relationship between the treatment class composition of dyads and the probability of observing association events over the course of the experiment (Supplementary Table 3). This model contained an indicator of dyad-type (same-class/different-class) and its interaction with a count of the number of association events that had occurred within the network (prior to time t). The interaction term was required to assess whether there was a change in the probability of observing same-class dyads associating, as compared to different-class dyads, over the course of the experiment. The main effect, when interpreted in conjunction with the interaction term mentioned above,

gave an estimate of the difference in the probability of observing same-class versus different-class dyads at the outset of the experiment.

Models 4 & 5: Differences in association rate attributable to properties of dyads (number of previous events, same/different treatment class) for affiliates (model 4) and non-affiliates (model 5).

REMs were fitted to determine whether the relationship between prior task experience and future association rate differed depending upon dyads' treatment class combination (same-class/different-class), and in turn whether this varied between affiliate and non-affiliate dyads. Prior task experience was split into three categories: no previous events (unfamiliar), between one and five previous events, and six or more previous events. Task experience was categorized to emphasise the distinction between unfamiliar (no previous association events) and familiar (at least one previous association event). For unfamiliar dyads, no difference in association rate attributable to the experimental treatment was to be expected, as individuals had not acquired information regarding how their partner's identity was linked to task pay-offs. However, for familiar dyads this learning effect was to be expected, so association rates of same-class dyads should exceed those of different-class dyads. Two categories of familiar dyad were used to capture the level of prior experience possessed by the majority of dyads (1-5 previous events; see Supplementary Fig. 1) and that of the minority of dyads that associated on many occasions (six or more previous events; see Supplementary Fig. 1). The interaction between prior experience level and treatment-class combination was included in the models to determine whether the relationship between prior experience and probability of observing association between members of a dyad in the future depended upon if the dyad members were incentivized (same-class) or disincentivized (different-class) for associating. To clarify model interpretation, the analysis was run separately for affiliate (Model 4; Supplementary Table 4) and non-affiliate (Model 5; Supplementary Table 5) dyads.

Models 6 & 7: The influence of the experimental treatment on dyadic coordination, for arrival latency (model 6) and association event duration (model 7).

Two REMs were fitted to investigate whether the treatment-class composition of dyads influenced coordination (Supplementary Tables 6 & 7). The two different measures used to quantify dyadic coordination were estimates of association event duration and arrival latency (both measured in seconds), where arrival latency was defined as the duration of time between the first and second members of a dyad arriving at a feeder to engage in an association event. Increased association event duration, and decreased arrival latency, was taken as indication of improvement in dyadic coordination for same-class dyads. Both REMs assessed the interaction of a dyadic coordination measure with an indicator of treatment-class composition (same-class/different-class). The main effect of the dyadic coordination measure (arrival latency in model 6, event duration in model 7) provided an indication of the likelihood of observing dyads, of any type, exhibiting different degrees of coordination. The interaction term indicated how this relationship between extent of dyadic coordination and likelihood of observing a dyad differed depending upon the treatment-class composition of dyads. The main effect of treatment-class composition provided an estimate of difference in the likelihood of observing same-class versus different-class dyads for low event durations or arrival latencies.

Model 8: Change in overlap in individuals' social neighbourhoods attributable to the experimental treatment.

This REM was fitted to assess whether any change in the likelihood of individuals sharing common task partners over the course of the experiment was attributable to the experimental treatment. The REM

contained the interaction of a term representing the number of events previously observed within the network (at the time of a given event) with two 'triadic closure' statistics (see Supplementary Note 5: Triadic closure statistics in Eventnet 0.5.2): the total number of individuals with which two members of the dyad in a given event at time *t* had both previously associated with (treatment-independent 'triadic closure'), and the total number of individuals with which two members of the dyad in a given event at time *t* had both previously associated with given that all individuals belonged to the same treatment class (treatment-dependent 'triadic closure'). Inclusion of the first statistic was necessary to provide an estimate of the baseline, treatment-independent, likelihood of individuals sharing common task partners. After controlling for treatment-independent changes in the baseline rate of association between individuals that shared common task partners, the treatment-dependent statistic then yielded an estimate of the effect of the experimental treatment on clustering by treatment class in the network (Supplementary Table 8).

3. Permutation procedure validation

A common constraint applied to data-stream permutation procedures is the randomization of events within spatio-temporal subsets of the data (e.g., randomization within combinations of day and feeder). The statistical power of subsequent permutation tests can be decreased if the permutation procedure is limited to acting on subsets of the dataset with few data points. In our dataset, there were 13 day-feeder combinations for which there was only a single data point, and for which permuted non-events could not be generated. As a consequence, approximately 2.5% of the permuted non-events did not differ in either source or target label from their corresponding unpermuted events (Supplementary Fig. 5a).

Permuting using swaps of the labels of pairs of events can, for some applications, be more efficient computationally than random shuffling of label sets, as verification of the validity of generated data can be performed following each individual operation, rather than following the randomization of an entire subset of the data. To ensure that the permutation procedure that we utilized was computationally tractable, we limited the number of shuffles attempted for each subset of the data. If this randomization limit was reached without a valid permutation of a given subset having been generated (defined as an edge list not containing loops, meaning that there were no events in which the same individual was listed as both the 'source' and the 'target'), then the dataset produced by the last attempt at shuffling the labels was taken and pairwise swaps were applied to it solely for the purpose of removing these loops (see Supplementary Fig. 2). Increasing the randomization limit increased the number of permuted subsets that could be successfully generated without employing swaps, but there were diminishing returns of increasing the limit (Supplementary Fig. 5b). Consequently, we decided to implement a limit of 10000 attempted randomizations per subset to achieve a compromise between robustness and computational tractability.

4. Visualization of REM output

To aid interpretation of REM output, predicted values were extracted from REMs and used to create figures. For a REM, predicted values represent the risk (i.e., hazard) of observing an individual or dyad with a given set of characteristics (i.e., predictor values) relative to an estimated baseline hazard. For REMs that contained categorical predictors, predicted values were calculated for every combination of factor levels, but for REMs with continuous predictors the number of predictions was capped (e.g., for Fig. 2, up to 10 same-class and 10 different-class participants).

The REMs we performed can be split into two categories: those that focussed on change over the course of the experiment and those that addressed hypotheses concerning prior experience. Predictions generated

from models that explicitly addressed change over the course of the experiment were compared to a baseline hazard specific to the time point (‘strata’; i.e., day) at which the prediction pertained (models 3 & 8, see Fig. 6c), whereas a single, sample-wide baseline hazard estimate was used for the other models (i.e., reference = “sample” in the predict.coxph function in R).

Predicted values were converted from relative risk multipliers (Incidence Rate Ratios) to estimates of hourly rate of association to aid figure interpretation. This was achieved by multiplying each predicted risk value by an appropriate estimate of the sample-wide baseline rate of association. These baseline estimates represented the estimated number of events per dyad, or per individual, per hour as calculated across all periods of the experiment. Rates were conditional upon the dyad or individual having participated in at least one event per hour, as the denominator used to calculate the rate was the number of dyads or individuals observed within each hour (rather than, for instance, the number that could have been observed per period). The following baseline estimates were used: number of successful events per individual per hour (used in Fig. 2), number of events per non-affiliate dyad per hour (see Fig. 4a), number of events per affiliate dyad per hour (see Fig. 4b), and number of events per (any) dyad per hour (see Fig. 6c).

Predictions were generated from each permuted REM, such that 10,000 predictions per combination of predictor levels was produced. From these distributions, median predicted values and prediction intervals were calculated for use in figures. For individual-level REMs (models 1 & 2), similar hypotheses were tested using multiple models. This was necessary to ensure that predictions suitable for use in figures were calculable. Consequently, for these models, a 97.5% prediction interval was calculated to mirror the 97.5% confidence intervals that were generated for these models (to account for multiple testing). For all other models a 95% prediction interval was used.

Supplementary Notes

1. Food preference test

To establish a high-quality and low-quality food reward, we assessed whether jackdaws (*Corvus monedula*) preferred to feed on a mealworm food mix rather than a standard mix that only consisted of grain. The mealworm mix contained an approximately 50/50 mixture of crushed, dried mealworms and grain. Equally sized (by volume) piles of the standard mix and mealworm mix were supplied at feeding tables during mid-to-late March 2017. Feeding table visits were filmed and the footage was later reviewed to determine whether foraging jackdaws' first food choice was the mealworm mix or the standard mix, or if they did not choose to feed at all. Of 357 recorded visits, jackdaws initially chose the mealworm mix on 250 occasions, chose the standard mix 97 times, and did not feed on 10 occasions. A binomial test confirmed that when jackdaws chose a food source, they displayed a significant preference for the mealworm mix (N.successes = 250, N.total = 347, expected probability of success = 0.5, two-sided test, $p < 0.001$). Consequently, the mealworm mix was used as the high-quality reward in the experiment and the standard mix was used as the low-quality reward.

2. Distribution of individuals' task activity during experimental days

During each experimental day, the task was active for an approximately four-hour period in the morning (beginning no earlier than 05:00 and ending no later than 12:00). If the full range of times across which the task was active (05:00 to 12:00) is split into half-hour windows (05:00-05:30 etc), then the mean number of half-hour windows a given individual was detected in was 4.64 (Standard Error (S.E.) = 2.66), with a range of 1-10 windows. For individuals that participated in at least 20 association events (both successes and failures, n=52, present in >90% of total interactions), this distribution of activity was greater (mean = 8.04, S.E. = 0.205, range = 4-10). Consequently, an individual was likely to have been observed at the feeders at multiple different stages of the task's daily active period. Finally, task interaction often occurred during social foraging. Splitting the full range of task active times into five-minute windows, for each of these windows in which one or more social association events were recorded a mean of 3.6 different individuals (with a maximum of 14) were observed.

3. Individual and dyad properties and treatment distributions

In total, 3117 association events were observed, of which 1727 (55.4%) were successful. Of these successful association events, 443 were between individuals that both belonged to treatment class A (of which 28 were between affiliates) and 1284 were between individuals that both belonged to treatment class B (of which 280 were between affiliates). In total, there were 648 associations between known affiliates (20.8% of the total number of associations), of which 308 were successful.

The total number of different dyads recorded was 751, of which 386 contained individuals in different treatment classes and 365 contained individuals in the same treatment class. Of the same-class dyads, 123 contained individuals that both belonged to treatment class A and 242 contained individuals that both belonged to treatment class B. A total of 139 individuals participated in at least one association event, of which 67 belonged to treatment class A and 72 belonged to treatment class B.

The majority of the 648 affiliate events involved mated pairs (580 total events involving seven dyads), while the remainder involved associations between parents and offspring (63 events across 10 dyads) or among siblings (5 events, 1 dyad). Supplementary Fig. 1 provides further detail of how the number of events scaled with the number of dyads for affiliates and non-affiliates.

4. Expected triad composition in experimental networks

When individuals are assigned to two distinct classes, two triad types can be observed in their social network (given that ties are undirected). The first type contains individuals that all belong to the same class, whereas the second contains individuals belonging to both classes. In total, there are eight different triads that can be formed in an undirected network with two node classes, of which two contain individuals that all belong to the same class (see Supplementary Fig. 4). Therefore, assuming that individuals mix with each other according to a random process, same-class-only triads should be three times less likely to be observed than multiple-class triads.

5. Triadic closure statistics in Eventnet 0.5.2

Triadic closure occurs when two individuals that have both previously been observed in an event with a common third individual then interact with each other. Triadic closure statistics can be devised to serve as simple indicators of the occurrence of triadic closure, or more detailed measures of the overall degree of convergence in two individuals' social neighbourhoods. We used a triadic closure statistic that provided the latter to track how the overlap between jackdaws' social circles changed over time, and whether change in this overlap depended upon the experimental conditions. The general form of the equation used to calculate this statistic in Eventnet 0.5.2 for a given event at time *t* was as follows:

$$triadic.closure.stat(A,B) = \sum_{i}^{n} [\max(attr(A,i), attr(i,A))] * [\max(attr(B,i), attr(i,B))]$$

where:

- A and B are the 'source' and 'target' of the given event
- 'attr' refers to an attribute that is updated with each successive event in the dataset. For example, an indicator of whether or not individuals A and B have previously been observed associating, or a count of the number of previous events that they have participated in together. In our analyses, the attribute was an indicator (True = 1; False = 0) of whether two individuals had associated before in a given source-target configuration.
- n refers to the total number of other individuals that both A and B have previously participated in an event with (i.e., common social partners), of which *i* is one of these individuals.

The triadic closure statistic we utilized was therefore a count of the number of common associates that a given dyad had at time *t*. Two versions of this statistic were calculated: a count of all common associates, regardless of treatment class, and a count of the number of common associates that belonged to the same class as both dyad members (i.e., same-class-only triads).

6. Limitations of permutation procedures based on constrained label swaps

Constraints are often imposed on a permutation procedure to prevent the generation of unrealistic permuted events. One such constraint that was applicable when permuting our data concerned the generation of loops. For example, an individual could not associate with itself, so in our dataset an association event could not have the same source and target labels. A valid permutation procedure should sample (approximately) uniformly at random from the set of possible permutations of the data given the constraints imposed. Consequently, an ideal permutation procedure approximates a Markov process, fulfilling the assumptions that every permutation of the data can be reached with a finite number of steps from any other permutation and that at each stage of the procedure the probability of transitioning from one permutation to another is equal to the reverse. We decided to primarily employ a shuffling of labels using the sample function in R as the basis of our permutation procedure to limit the use of label swaps, as the use of successive constrained swaps can violate the above conditions (see Supplementary Fig. 6).

Supplementary Figures

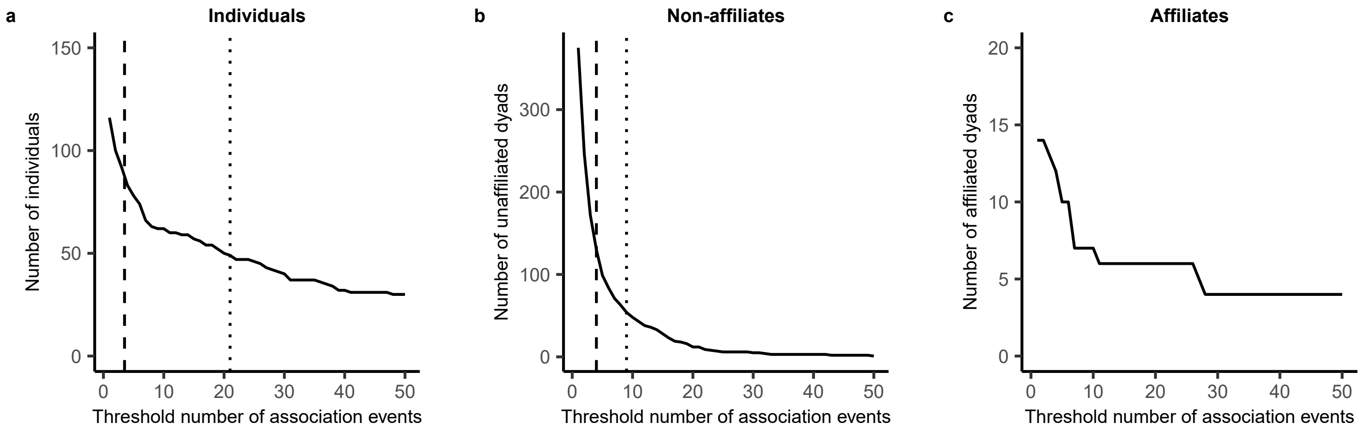

Supplementary Figure 1 | Distribution of overall task experience amongst individuals and dyads. Illustration of the number of (**a**) individuals, (**b**) non-affiliate dyads and (**c**) affiliated dyads that participated in greater than the specified number of association events. Vertical lines indicate the number of association events that at least 100 (dashed line) or 50 (dotted line) individuals or dyads participated in.

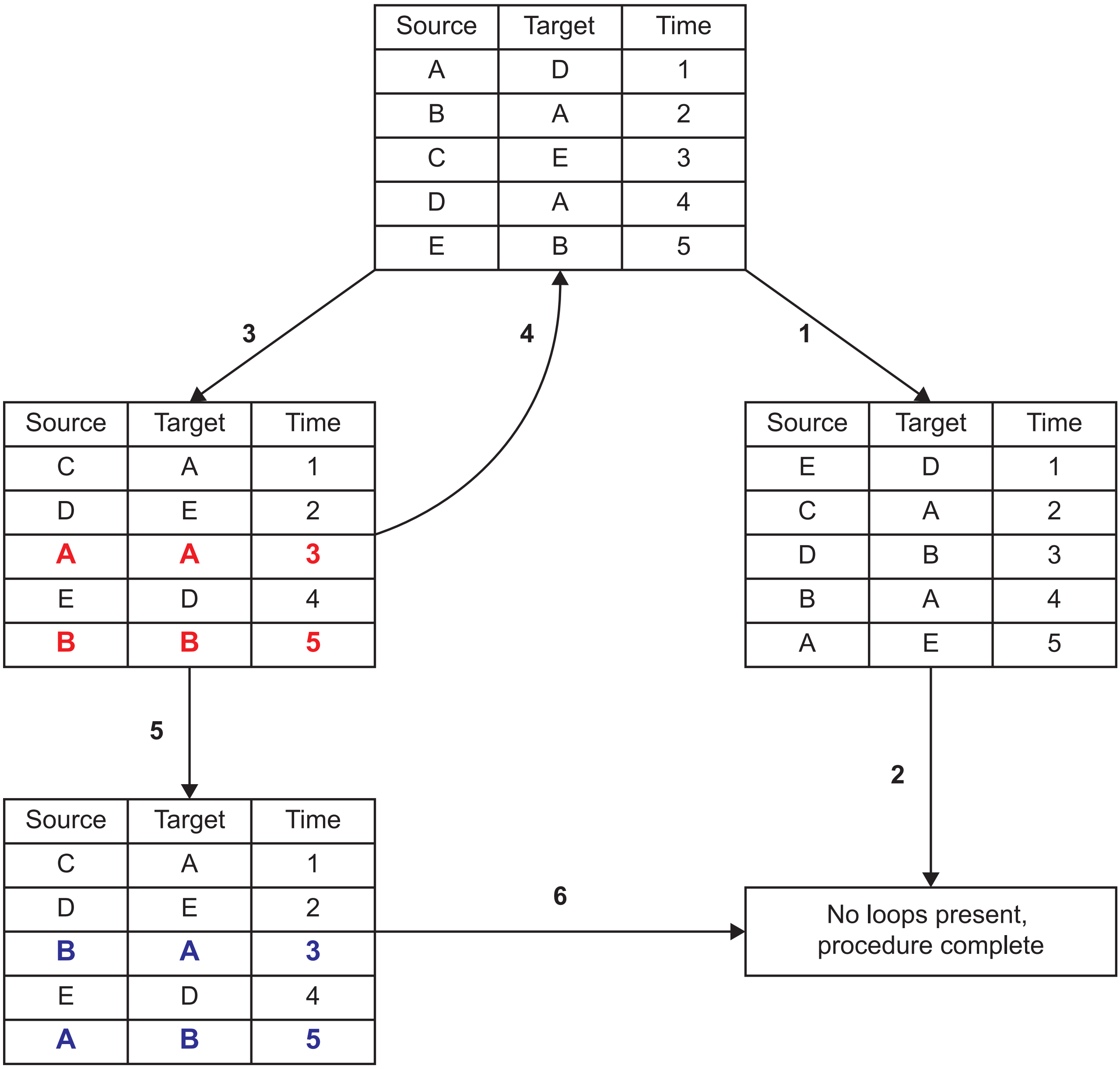

Supplementary Figure 2 | An example of a permutation procedure composed of label shuffling and pairwise label swaps as applied to a REM dataset. For each subset of the unpermuted data (Top Table), the ID labels from each event (Source and Target) are randomized in an unconstrained, unsupervised fashion (random shuffle). In this example, the Source and Target columns are randomized independently (i.e., without mixing of 'source' and 'target' labels). Unconstrained randomization can generate a dataset in which all events contain valid combinations of source and target labels (1), such that the randomization of a given subset of the data is completed without pairwise label-swapping (2). Alternatively, a permuted dataset that contains invalid source-target label combinations (red) can be generated (3). In this experiment, individuals could not associate with themselves, so these 'loop' instances must be removed. If the number of attempts at generating a valid permuted dataset from the current subset has not reached a certain threshold (see Supplementary Methods: Validation of permutation procedure and Supplementary Fig. 5 for details of threshold selection), and a dataset without loops has not been generated, then randomization via unconstrained shuffling of labels is repeated (4). If the randomization limit is reached without a valid dataset having been generated, then the most recent generated dataset is examined to locate invalid instances and labels are swapped between these instances in a pairwise fashion (5) to produce valid events from loops (blue). Once all invalid instances have been removed from this permuted dataset, the procedure is complete for the current subset (6).

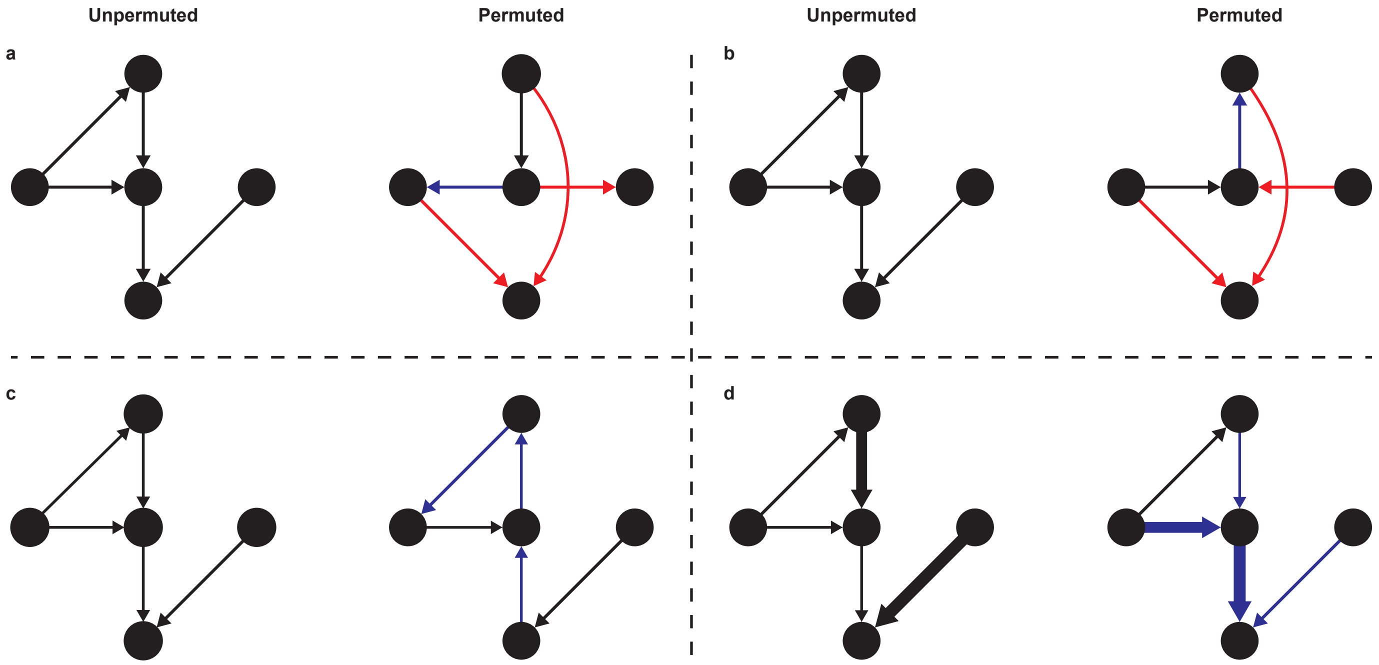

Supplementary Figure 3 | Illustration of the four ways that data-stream permutation can be applied to non-bipartite REM datasets. In each case, the initial unpermuted network is displayed, accompanied by the result of applying the given type of permutation to the REM dataset. Node (circle) and edge (arrow) colours signify change in the properties of existing nodes or edges (blue) or creation of new nodes or edges (red) caused by the permutation procedure. Source and target labels can be randomized non-independently (**a**), such that a permuted event can contain any combination of the available 'source' or 'target' (see Supplementary Table 10 for definitions) labels observed within the given sampling period. Non-independent source and target label randomization can generate new edges, alter the direction of existing edges and alter node properties (each node's total number of events is preserved, but the balance of events as source or target can change). Source and target labels can be randomized independently (**b**), whereby source labels can only be exchanged with other source labels and target labels can only be exchanged with target labels. This process preserves node properties, as each individual's activity as both a source and as a target, rather than just overall activity, remains fixed. Swaps of source labels with their corresponding target labels only randomizes edge direction and does not create or delete edges, so preserving all observed dyadic interactions (**c**), but altering node properties (number of events as source versus target). Similarly, for weighted REM data, edge weights can be randomized without altering the underlying network structure (**d**), so edge weights are permuted without the procedure creating or deleting edges, altering edge direction, or altering node properties.

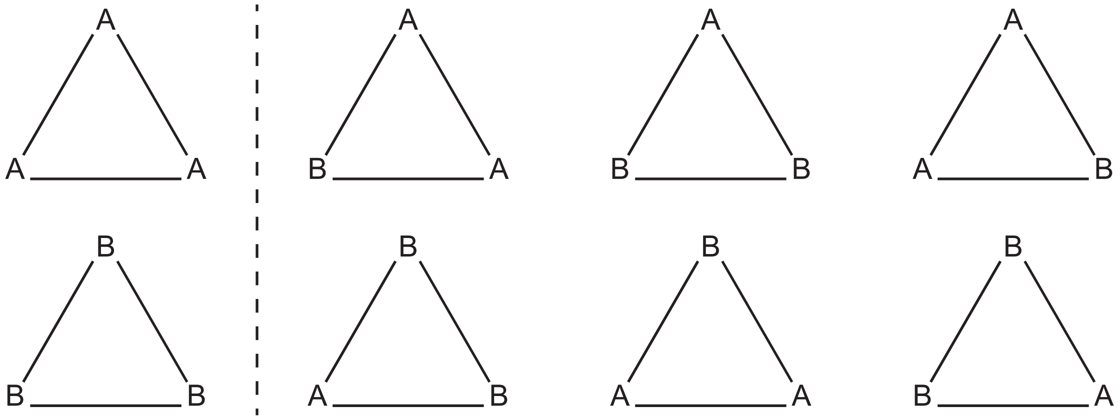

Supplementary Figure 4 | The eight triad configurations that can be formed between individuals that belong to two distinct classes (A & B). When each tie within a triad is formed in turn, the interaction of two individuals that belong to the same class and have both previously interacted with a common third member of the same class results in the formation of a same-class-only triad, of which there are two forms (AAA & BBB). Six other mixed composition triads, in which only one of the three ties is between members of the same class, can also be observed if order of tie formation within the triad is accounted for.

a

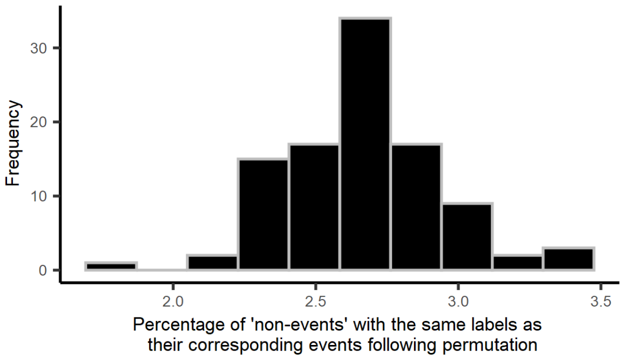

b

Maximum number of randomizations	Minimum %	Maximum %
1	9.6	24.1
10	30.1	49.4
100	60.2	72.3
1000	77.1	86.7
10000	86.7	95.2
100000	92.8	96.4

Supplementary Figure 5 | Permutation procedure diagnostics. Histogram of the percentage of REM 'non-events' (see Supplementary Table 10: Glossary of common REM terms), generated from non-independent permutation of source and target labels, that have both the same source and target labels as their corresponding observed events in the dataset (**a**). The distribution contains the percentage values for 100 permuted datasets and shows the extent to which the permuted datasets were likely to remain unchanged from the original, unpermuted data. The limit of the number of attempted randomizations applied to each subset of the data affects the percentage of the data that can be randomized without using an additional procedure, such as pairwise swaps, to remove loops (**b**). The minimum and maximum percentage of randomization success (i.e., the number of subsets successfully randomized, without resorting to the use of swaps, divided by the total number of subsets), as calculated from 100 permuted datasets, is displayed for a range of subset randomization limits.

a

Source	Target	Time
A	B	t1
B	A	t2

b

Source	Target	Time
B	A	t1
A	B	t2

c

Source	Target	Time
A	B	t1
B	D	t2
C	A	t3

d

Source	Target	Time
A	B	t1
C	D	t2
B	A	t3

Supplementary Figure 6 | The impact of constraining label swaps on the generation of permuted datasets. By utilizing serial swaps of either source or target labels (with other source or target labels respectively), constrained such that events containing the same source and target label cannot be generated by the procedure (i.e., loops not allowed), applying a single swap to the original data (**a**) cannot generate certain valid permutations of the data (**b**). Similarly, the probability of transitioning between different permutations of the data is not equal. Following a swap, the number of potential future swaps can differ from the number of possible swaps at the prior stage of the procedure. For example, prior to any swaps occurring two valid swaps are available in a dataset (**c**: pre-swap, valid swaps - Source 'B' with Source 'C' or Target 'D' with Target 'A'), but following a particular swap (Source 'B' with Source 'C') four valid swaps are now possible in the next iteration of the procedure (**d**: post-swap, valid swaps - Source 'A' with Source 'C', Source 'C' with Source 'B', Target 'B' with Target 'D' and Target 'D' with Target 'A').

Supplementary Tables

Supplementary Table 1: Relationship of task performance with individuals' previous number of successful events with familiar participants (at least one previous association) and number of unsuccessful events with familiar participants, for individuals that joined (rather than initiated) paired feeding events at feeders.

**REM term**	**95% Confidence Interval of coefficient values (Lower Bound)**	**Median coefficient value**	**95% Confidence Interval of coefficient values (Upper Bound)**	**95% Confidence Interval of Incidence Rate Ratio (Lower Bound)**	**Median Incidence Rate Ratio**	**95% Confidence Interval of Incidence Rate Ratio (Upper Bound)**	**Significant?**
target_repeat_success_count	0.00108	0.00149	0.00189	1.0011	1.0015	1.0019	**Y**
target_repeat_fail_count	-0.00434	-0.00312	-0.00183	0.996	0.997	0.998	**Y**
target_repeat_success_count:target_repeat_fail_count	-1.71E-05	-4.87E-06	7.60E-06	1.00	1.00	1.0000076	N

Glossary for Supplementary Table 1:

**Term**	**Type**	**Level of Analysis**	**Explanation/Question**
target_repeat_success_count	Main effect	Individual	An individual's number of previous successful events with participants with which they were already familiar (had engaged in at least one association with). Were individuals that had a greater number of repeated associations with familiar same-class associates more likely to be observed in successful events in the future?
target_repeat_fail_count	Main effect	Individual	An individual's number of previous unsuccessful events with participants with which they were already familiar (had engaged in at least one association with). Were individuals that had a greater number of repeated associations with familiar different-class associates less likely to be observed in successful events in the future?
target_repeat_success_count:target_repeat_success_count	Interaction	Individual	Interaction between counts of repeated successes and repeated failures. Required to determine whether the relationship between past and future success was dependent upon quantity of past failures.

Supplementary Table 2: Relationship of task performance with individuals' number of same-class partners with which they had associated on multiple occasions and number of different-class partners with which they had associated on multiple occasions, for individuals that joined (rather than initiated) paired feeding events at feeders.

REM term	95% Confidence Interval of coefficient values (Lower Bound)	Median coefficient value	95% Confidence Interval of coefficient values (Upper Bound)	95% Confidence Interval of Incidence Rate Ratio (Lower Bound)	Median Incidence Rate Ratio	95% Confidence Interval of Incidence Rate Ratio (Upper Bound)	Significant?
repeat_success_deg	0.00269	0.0146	0.0270	1.0027	1.015	1.027	**Y**
repeat_fail_deg	-0.0511	-0.0404	-0.0293	0.950	0.960	0.971	**Y**
repeat_success_deg:repeat_fail_deg	0.00105	0.00212	0.00324	1.0011	1.0021	1.0032	**Y**

Glossary for Supplementary Table 2:

**Term**	**Type**	**Level of Analysis**	**Explanation/Question**
repeat_success_deg	Main effect	Individual	Count of the number of same-class task partners with which an individual has associated on multiple occasions. Were individuals with a greater number of same-class partners with which they had associated repeatedly more likely to be observed engaging in successful events in the future?
repeat_fail_deg	Main effect	Individual	Count of the number of different-class task partners with which an individual has associated on multiple occasions. Were individuals with a greater number of different-class partners with which they had associated repeatedly less likely to be observed engaging in successful events in the future?
repeat_success_deg :repeat_fail_deg	Interaction	Individual	Interaction between counts of number of same-class and different-class participants with which an individual had associated on multiple occasions. Required to assess whether any link between number of past familiar same-class partners and future success was dependent upon an individual's propensity to associate with different-class participants on multiple occasions. Was an individual with a given number of same-class partners with which it had associated on multiple occasions more likely to be observed engaging in successful events in the future if it had few, as compared to many, different-class individuals with which it had associated repeatedly?

Supplementary Table 3: Change in association rate over the course of the experiment for different dyad types (same/different treatment class).

REM term	95% Confidence Interval of coefficient values (Lower Bound)	Median coefficient value	95% Confidence Interval of coefficient values (Upper Bound)	95% Confidence Interval of Incidence Rate Ratio (Lower Bound)	Median Incidence Rate Ratio	95% Confidence Interval of Incidence Rate Ratio (Upper Bound)	Significant?
same_group	-0.250	-0.167	-0.0869	0.779	0.846	0.917	**Y**
same_group:network.num.coop (per 1000 network events)	0.143	0.190	0.237	1.15	1.21	1.27	**Y**

Glossary for Supplementary Table 3:

Term	Type	Level of Analysis	Explanation/Question
same_group	Main effect	Dyad	At the outset of the experiment, did dyads in the same treatment class differ in association rate from dyads in which members belonged to different treatment classes?
same_group:network.num.coop	Interaction	Dyad	Did the rate at which same-class dyads were observed associating – as compared to the rate at which different-class dyads were observed (i.e., relative rate) – change as the experiment progressed? Coefficient values and incidence rates are scaled (given per 1000 network events, rather than per event) to aid interpretation.

Supplementary Table 4: Differences in association rate amongst affiliated dyads attributable to dyad properties (number of previous events, same/different treatment class).

**REM term**	**95% Confidence Interval of coefficient values (Lower Bound)**	**Median coefficient value**	**95% Confidence Interval of coefficient values (Upper Bound)**	**95% Confidence Interval of Incidence Rate Ratio (Lower Bound)**	**Median Incidence Rate Ratio**	**95% Confidence Interval of Incidence Rate Ratio (Upper Bound)**	**Significant?**
same_group	-0.557	-0.248	0.0604	0.573	0.780	1.062	N
dyad.coop.catFAMILIAR_1TO5	0.961	1.20	1.45	2.62	3.32	4.26	**Y**
dyad.coop.catFAMILIAR_6PLUS	3.049	3.26	3.47	21.1	26.1	32.3	**Y**
same_group:dyad.coop.catFAMILIAR_1TO5	-0.160	0.242	0.637	0.852	1.27	1.89	N
same_group:dyad.coop.catFAMILIAR_6PLUS	-0.452	-0.116	0.218	0.636	0.891	1.24	N

Glossary for Supplementary Table 4:

**Term**	**Type**	**Level of Analysis**	**Explanation/Question**
dyad.coop.cat	Main effect	Dyad	Did the number of previous association events engaged in by a dyad composed of affiliates affect the probability of observing the dyad associate in the future? Three categories: “ARE_UNFAMILIAR” (no previous events; reference level), “FAMILIAR_1TO5” (between 1 and 5 previous events), “FAMILIAR_6PLUS” (6 or more previous events).
same_group	Main effect	Dyad	Did affiliate dyads in the same treatment class differ in association rate from affiliate dyads in which members belonged to different treatment classes?
same_group:dyad.coop.cat	Interaction	Dyad	Was there a difference in the relationship between prior task experience and probability of observing a dyad associate in the future for same-class as compared to different-class affiliate dyads?

Supplementary Table 5: Differences in association rate amongst non-affiliated dyads attributable to dyad properties (number of previous events, same/different treatment class).

REM term	95% Confidence Interval of coefficient values (Lower Bound)	Median coefficient value	95% Confidence Interval of coefficient values (Upper Bound)	95% Confidence Interval of Incidence Rate Ratio (Lower Bound)	Median Incidence Rate Ratio	95% Confidence Interval of Incidence Rate Ratio (Upper Bound)	Significant?
same_group	-0.07	0.0182	0.105	0.932	1.018	1.11	N
dyad.coop.catFAMILIAR_1TO5	0.212	0.297	0.378	1.24	1.35	1.46	**Y**
dyad.coop.catFAMILIAR_6PLUS	0.0193	0.111	0.207	1.019	1.12	1.23	**Y**
same_group:dyad.coop.catFAMILIAR_1TO5	0.0548	0.176	0.298	1.056	1.19	1.35	**Y**
same_group:dyad.coop.catFAMILIAR_6PLUS	0.155	0.290	0.423	1.17	1.34	1.53	**Y**

Glossary for Supplementary Table 5:

Term	Type	Level of Analysis	Explanation/Question
dyad.coop.cat	Main effect	Dyad	Did the number of previous association events engaged in by a dyad composed of non-affiliates affect the probability of observing the dyad associate in the future? Three categories: “ARE_UNFAMILIAR” (no previous events; reference level), “FAMILIAR_1TO5” (between 1 and 5 previous events), “FAMILIAR_6PLUS” (6 or more previous events).
same_group	Main effect	Dyad	Did non-affiliate dyads in the same treatment class differ in association rate from non-affiliate dyads in which members belonged to different treatment classes?
same_group:dyad.coop.cat	Interaction	Dyad	Was there a difference in the relationship between prior task experience and probability of observing a dyad associate in the future for same-class as compared to different-class non-affiliate dyads?

Supplementary Table 6: Differences in arrival latency preceding association events for dyads of different types (same/different treatment class).

REM term	95% Confidence Interval of coefficient values (Lower Bound)	Median coefficient value	95% Confidence Interval of coefficient values (Upper Bound)	95% Confidence Interval of Incidence Rate Ratio (Lower Bound)	Median Incidence Rate Ratio	95% Confidence Interval of Incidence Rate Ratio (Upper Bound)	Significant?
WEIGHT	-0.00302	0.00180	0.00718	0.997	1.0018	1.0072	N
same_group	-0.0275	0.0207	0.0725	0.973	1.021	1.075	N
WEIGHT:same_group	-0.0105	-0.00295	0.00394	0.990	0.997	1.0039	N

Glossary for Supplementary Table 6:

**Term**	**Type**	**Level of Analysis**	**Explanation/Question**
WEIGHT	Main effect	Dyad	Time between the arrival at the feeder of the two participants in an association event, in seconds. Did the likelihood of observing a dyad (of any type) change with increasing arrival latency?
same_group	Main effect	Dyad	When arrival latencies were short (i.e., for arrival latency = 0 seconds), was there a difference in the likelihood of observing same- versus different-class dyads?
WEIGHT:same_group	Interaction	Dyad	Did the relationship between arrival latency and likelihood of observation depend upon whether members of a dyad belonged to the same treatment class or different treatment classes?

Supplementary Table 7: Differences in association event duration for dyads of different types (same/different treatment class).

REM term	95% Confidence Interval of coefficient values (Lower Bound)	Median coefficient value	95% Confidence Interval of coefficient values (Upper Bound)	95% Confidence Interval of Incidence Rate Ratio (Lower Bound)	Median Incidence Rate Ratio	95% Confidence Interval of Incidence Rate Ratio (Upper Bound)	Significant?
WEIGHT	-0.000516	0.00147	0.00369	0.999	1.0015	1.0037	N
same_group	-0.00859	0.0232	0.0560	0.991	1.024	1.058	N
WEIGHT:same_group	-0.00609	-0.00251	0.000922	0.994	0.997	1.00092	N

Glossary for Supplementary Table 7:

Term	Type	Level of Analysis	Explanation/Question
WEIGHT	Main effect	Dyad	How did the likelihood of observing a dyad (of any type) change given different association event durations (in seconds)?
same_group	Main effect	Dyad	When event durations were short (i.e., for event duration = 0 seconds), was there a difference in the likelihood of observing same- versus different-class dyads?
WEIGHT:same_group	Interaction	Dyad	Did the relationship between event duration and likelihood of observation depend upon whether members of a dyad belonged to the same treatment class or different treatment classes?

Supplementary Table 8: Change in frequency of association between individuals that shared common associates over the course of the experiment.

REM term	95% Confidence Interval of coefficient values (Lower Bound)	Median coefficient value	95% Confidence Interval of coefficient values (Upper Bound)	95% Confidence Interval of Incidence Rate Ratio (Lower Bound)	Median Incidence Rate Ratio	95% Confidence Interval of Incidence Rate Ratio (Upper Bound)	Significant?
triadic.closure.coop (per associate)	-0.0000295	0.00595	0.0118	0.99997	1.0060	1.012	N
success.closure (per associate)	-0.0469	-0.0285	-0.0105	0.954	0.972	0.990	**Y**
triadic.closure.coop:network.num.coop (per associate, per 1000 network events)	-0.00507	-0.00220	0.000708	0.995	0.998	1.00071	N
success.closure:network.num.coop (per associate, per 1000 network events)	0.00530	0.0145	0.0241	1.0053	1.015	1.024	**Y**

Glossary for Supplementary Table 8:

Term	Type	Level of Analysis	Explanation/Question
triadic.closure.coop	Main effect	Network	At the beginning of the experiment, were individuals that shared many common associates (regardless of their treatment class) more likely to be observed than individuals with few common associates?
success.closure	Main effect	Network	At the beginning of the experiment, were individuals belonging to the same treatment class more likely to be observed associating if they had many same-class common associates than if they had few same-class common associates?
triadic.closure.coop:network.num.coop	Interaction	Network	As the experiment progressed, did the relative likelihood of observing dyads with many common associates (of either treatment class), as compared to few common associates, change?
success.closure:network.num.coop	Interaction	Network	As the experiment progressed, did the relative likelihood of observing dyads (in which both members belonged to the same class) with many same-class common associates, as compared to few same-class common associates, change?

Supplementary Table 9 | Transcript of events recorded by a 'Darwin Board' microcomputer during the period in which Supplementary Movie 1 was recorded. The arrival and departure of each individual was logged in addition to the outcome of dual-feeding events. Supplementary Movie 1 is available from Figshare (https://figshare.com/collections/Cornish-Jackdaws/6723399).

**Timestamp**	**Event**	**RFID-Tag code**	**Time (in video)**	**Leg ring code**	**Class**	**Description of event**
190605061007	START_EXPERIMENT					
190605073117	TAG_DEPARTED_B	01c4ca00	00:00:29	MYBP	B	Solo feeding bird (low-quality - Left Door) departs
190605073120	TAG_ARRIVED_B	0102ae00	00:00:30	WMWY	A	
190605073121	SOLO_SUCCESS	0102ae00		WMWY	A	Solo visit yielding low quality (Left Door) reward
190605073124	TAG_ARRIVED_A	01c4ca00	00:00:38	MYBP	B	
190605073125	STARTCOOP_FAIL	0102ae00 & 01c4ca00		MYBP & WMWY	B & A	Incompatible pair results in failure and no reward
190605073126	ENDCOOP_FAIL	0102ae00 & 01c4ca00		MYBP & WMWY	B & A	
190605073126	TAG_DEPARTED_B	0102ae00		WMWY	A	
190605073127	TAG_ARRIVED_B	0102ae00		WMWY	A	
190605073128	STARTCOOP_FAIL	01c4ca00 & 0102ae00		MYBP & WMWY	B & A	
190605073129	TAG_DEPARTED_A	01c4ca00	00:00:45	MYBP	B	
190605073130	ENDCOOP_FAIL	0102ae00_		WMWY_	A	
190605073131	TAG_DEPARTED_B	0102ae00	00:00:46	WMWY	A	
190605073133	TAG_ARRIVED_A	0102ae00	00:00:47	WMWY	A	
190605073133	SOLO_FAIL	0102ae00		WMWY	A	Solo visit to feeder in a lockout period - no reward
190605073136	TAG_DEPARTED_A	0102ae00		WMWY	A	
190605073138	TAG_ARRIVED_A	0102ae00		WMWY	A	
190605073139	SOLO_FAIL	0102ae00		WMWY	A	
190605073142	TAG_DEPARTED_A	0102ae00	00:00:55	WMWY	A	
190605073147	TAG_ARRIVED_A	01c4ca00	00:01:00	MYBP	B	
190605073147	SOLO_FAIL	01c4ca00		MYBP	B	Solo visit to feeder in a lockout period - no reward
190605073151	TAG_DEPARTED_A	01c4ca00	00:01:05	MYBP	B	
190605073152	TAG_ARRIVED_B	01c4ca00	00:01:05	MYBP	B	
190605073153	SOLO_FAIL	01c4ca00		MYBP	B	Solo visit to feeder in a lockout period - no reward
190605073203	TAG_ARRIVED_A	01043c00	00:01:14	OVRM	B	
190605073204	STARTCOOP_SUCCESS	01c4ca00 & 01043c00		MYBP & OVRM	B & B	Compatible pair results in success and high quality (Right Door) reward

Supplementary Table 9. Continued

Timestamp	Event	RFID-Tag code	Time (in video)	Leg ring code	Class	Description of event
190605073205	TAG_DEPARTED_A	01043c00	00:01:17	OVRM	B	
190605073205	TAG_ARRIVED_A	01fe7600	00:01:17	YZMOR	A	
190605073206	STARTCOOP_FAIL	01c4ca00 & 01fe7600		MYBP & YZMOR	B & A	Incompatible pair results in failure and no reward
190605073206	ENDCOOP_FAIL	01c4ca00 & 01fe7600		MYBP & YZMOR	B & A	
190605073206	TAG_DEPARTED_B	01c4ca00	00:01:18	MYBP	B	
190605073208	TAG_DEPARTED_A	01fe7600	00:01:17	YZMOR	A	
190605073210	TAG_ARRIVED_A	01009100	00:01:23	RZGGM	B	
190605073211	SOLO_FAIL	01009100		RZGGM	B	
190605073212	TAG_ARRIVED_B	01fe7600	00:01:25	YZMOR	A	
190605073212	STARTCOOP_FAIL	01009100 & 01fe7600		RZGGM & YZMOR	B & A	
190605073213	TAG_ARRIVED_B	01c4ca00	00:01:27	MYBP	B	
190605073214	STARTCOOP_SUCCESS	01009100 & 01c4ca00		RZGGM & MYBP	B & B	Compatible pair results in success and high quality (Right Door) reward
190605073214	TAG_DEPARTED_A	01009100	00:01:37	RZGGM	B	
190605073217	ENDCOOP_SUCCESS	01c4ca00_		MYBP_	B	

Supplementary Table 10: Glossary of Relational-Event Model (REM) terminology.

**Term**	**Model**	**Explanation/Question**
Source	ALL	The individual that can be classed as the ‘sender’ in a dyadic event. In our analyses, the individual engaging in a given association event that arrived at the feeder first was labelled as the ‘source’.
Target	ALL	The individual that can be classed as the ‘receiver’ in a dyadic event. In our analyses, the individual engaging in a given association event that arrived at the feeder second (i.e., after the 'source') was labelled as the ‘target’.
Relational event	ALL	An event occurring between two entities at a specific point in time. Directionality is implied (i.e., events directed from one entity (the ‘source’) to another (the ‘target’)), but is not strictly necessary for the purposes of analysis. It can, for example, be just a convention (e.g., focal individuals labelled as sources, non-focal as targets) in cases where events lack an obvious direction of action between participants (e.g., social associations).
Time-varying covariate	ALL	Covariate which changes in value over time (i.e., throughout the span of a longitudinal dataset). In Eventnet, ‘statistics’ derived from ‘attributes’ (see Eventnet tutorials available at https://github.com/juergenlerner/eventnet) are evaluated at each time step in the dataset.
‘non-event’	ALL	An instance generated by the user, for example via a permutation procedure, for the purposes of hypothesis testing. Conceptually, ‘non-events’ can be viewed as the ‘expected’ data to which the observed events are to be compared. At least one ‘non-event’ is required for each event in a REM dataset, and each event is compared to its corresponding ‘non-event(s)’.
Conditioned on Source/Target	Models 1 & 2	For individual-level REM analyses, hypotheses must be tested for either the individual that initiated an event (the Source) or the individual that received/joined the event (the Target). To prevent variation in the properties of individuals belonging to the other category (i.e., Sources if the analysis wishes to focus on Targets) from affecting results, the ‘non-events’ are ‘conditioned’, meaning that every non-event has the same label (in this example Source labels) as its corresponding event.

Supplementary Table 11 | Influence of the concatenation and filtering processes on the balance of number and duration of successful and unsuccessful association events. The first row contains values from the raw data, pre-processing, whilst the second entry contains values from the processed dataset, following concatenation of adjacent association events and removal of events.

**Concatenation threshold (s)**	**Number of successes**	**Number of failures**	**Duration of successes (s)**	**Duration of failures (s)**	**Proportion success (Event count)**	**Proportion success (Event duration)**
0 (i.e., no concatenation)	4265	3058	11349	10501	0.582	0.519
15	1727	1390	16338	12407	0.554	0.568
