# Supplementary material for: Wild jackdaws can selectively adjust their social associations while preserving valuable long-term relationships
Source: Nat Commun. 2023 Sep 11;14:5103. doi: 10.1038/s41467-023-40808-7 (PMC10495349; doi:10.1038/s41467-023-40808-7)
Supplement: Supplementary file 3 — Description of Additional Supplementary Files [file 41467_2023_40808_MOESM3_ESM.pdf]

Description of Additional Supplementary Files

Supplementary Movie 1

Description: Video clip illustrating the dynamic nature of social activity during engagement with the dual-feeder task. Successful events (associations between compatible partners yielding high value food rewards) occur at 01:14 and 01:27 (see Supplementary Table 2 in the Supplementary Material for a complete transcript of events). Multiple different individuals can be seen and heard in the vicinity of the task throughout.
