# Supplementary material for: Wild jackdaws can selectively adjust their social associations while preserving valuable long-term relationships
Source: Nat Commun. 2023 Sep 11;14:5103. doi: 10.1038/s41467-023-40808-7 (PMC10495349; doi:10.1038/s41467-023-40808-7)
Supplement: Supplementary file 5 — Reporting Summary [file 41467_2023_40808_MOESM5_ESM.pdf]

nature portfolio

Corresponding author(s): Michael Kings
Josh Arbon
Alex Thornton

Last updated by author(s): Jul 3, 2023

Reporting Summary

Nature Portfolio wishes to improve the reproducibility of the work that we publish. This form provides structure for consistency and transparency in reporting. For further information on Nature Portfolio policies, see our Editorial Policies and the Editorial Policy Checklist.

Statistics

For all statistical analyses, confirm that the following items are present in the figure legend, table legend, main text, or Methods section.

n/a	Confirmed	
☐	☒	The exact sample size (*n*) for each experimental group/condition, given as a discrete number and unit of measurement
☐	☒	A statement on whether measurements were taken from distinct samples or whether the same sample was measured repeatedly
☐	☒	The statistical test(s) used AND whether they are one- or two-sided<br>*Only common tests should be described solely by name; describe more complex techniques in the Methods section.*
☐	☒	A description of all covariates tested
☐	☒	A description of any assumptions or corrections, such as tests of normality and adjustment for multiple comparisons
☐	☒	A full description of the statistical parameters including central tendency (e.g. means) or other basic estimates (e.g. regression coefficient) AND variation (e.g. standard deviation) or associated estimates of uncertainty (e.g. confidence intervals)
☐	☒	For null hypothesis testing, the test statistic (e.g. *F*, *t*, *r*) with confidence intervals, effect sizes, degrees of freedom and *P* value noted<br>*Give P values as exact values whenever suitable.*
☒	☐	For Bayesian analysis, information on the choice of priors and Markov chain Monte Carlo settings
☐	☒	For hierarchical and complex designs, identification of the appropriate level for tests and full reporting of outcomes
☒	☐	Estimates of effect sizes (e.g. Cohen's *d*, Pearson's *r*), indicating how they were calculated

Our web collection on statistics for biologists contains articles on many of the points above.

Software and code

Policy information about availability of computer code

Data collection	The C code for 'Darwin Board' microcomputer programming and R code used for treatment class assignment is available from Github (see https://github.com/mkings-220920/Cornish-Jackdaws). Note, the C code uses proprietary libraries that will not be made publicly available.
Data analysis	Eventnet 0.5.2 and Eventnet tutorials are available from Github (https://github.com/juergenlerner/eventnet). The R scripts used for data processing and analysis are also available from Github (https://github.com/mkings-220920/Cornish-Jackdaws). The following R packages were used for data processing, analysis or visualization: ggplot2 (version 3.2.1), survival (version 2.44), scales (version 1.0.0).

For manuscripts utilizing custom algorithms or software that are central to the research but not yet described in published literature, software must be made available to editors and reviewers. We strongly encourage code deposition in a community repository (e.g. GitHub). See the Nature Portfolio guidelines for submitting code & software for further information.

Data

Policy information about availability of data

All manuscripts must include a data availability statement. This statement should provide the following information, where applicable:

- Accession codes, unique identifiers, or web links for publicly available datasets
- A description of any restrictions on data availability
- For clinical datasets or third party data, please ensure that the statement adheres to our policy

The data that support the findings of this study can be accessed from Figshare (https://figshare.com/collections/Cornish-Jackdaws/6723399). We provide the raw .csv files downloaded from the task apparatus after each session, the processed data combined into a single .csv file, the permuted REM datasets, and the data

required to reproduce the figures. Further documentation on the uses of each dataset type and the data analysis workflow are available (https://github.com/mkings-220920/Cornish-Jackdaws/blob/main/Workflow%20diagram.pptx) as is an explanation of the structure and content of the processed REM datasets (https://github.com/mkings-220920/Cornish-Jackdaws/blob/main/Data/REM%20dataset%20structure%20and%20content.docx).

Research involving human participants, their data, or biological material

Policy information about studies with human participants or human data. See also policy information about sex, gender (identity/presentation), and sexual orientation and race, ethnicity and racism.

Reporting on sex and gender	Not applicable
Reporting on race, ethnicity, or other socially relevant groupings	Not applicable
Population characteristics	Not applicable
Recruitment	Not applicable
Ethics oversight	Not applicable

Note that full information on the approval of the study protocol must also be provided in the manuscript.

Field-specific reporting

Please select the one below that is the best fit for your research. If you are not sure, read the appropriate sections before making your selection.

☐ Life sciences ☐ Behavioural & social sciences ☒ Ecological, evolutionary & environmental sciences

For a reference copy of the document with all sections, see nature.com/documents/nr-reporting-summary-flat.pdf

Ecological, evolutionary & environmental sciences study design

All studies must disclose on these points even when the disclosure is negative.

Study description

The experiment used automated social coordination tasks, constructed using Radio-Frequency Identification (RFID) data-loggers and 'Darwin Board' microcomputers, to examine partner-choice dynamics in a group of wild jackdaws (Corvus monedula). Data-logger data enabled the determination of the identities of task participants and the timing and duration of paired foraging events. To manipulate the value of social foraging associations, participants were assigned at random to one of two experimental treatment classes, and the combination of participants' classes determined the food rewards accessible during paired foraging at the task apparatus. The study used data collected during weekday mornings across a breeding season (April - July 2019), in total comprising 3117 paired foraging events. Relational Event Models (REMs), which are designed for analysis of social network dynamics, were used to analyze how individual, dyad and network characteristics changed over time in response to the experimental treatment. The combination of treatment classes of the participants (same-class, different-class) and a categorization of the nature of a pairing's pre-existing relationship (affiliates, non-affiliates) featured in all models. In each of the models, the response term represented the rate at which an individual, dyad or grouping with given characteristics was estimated to be observed relative to an appropriate reference level (e.g. same-class versus different-class). In all models, this output was the difference between observed rates and expected rates as estimated from permuted data. Permutation procedures were utilized to produce suitable null models for hypothesis-testing.

Research sample

Jackdaws provide an ideal system to study the cognitive basis and group-level consequences of social decision-making because they live in groups featuring stable relationships between long-term affiliates as well as frequent interactions between unaffiliated individuals outside of these relationships. We used an automated social coordination task to examine partner-choice decision-making in a wild population of ringed jackdaws. The majority of individuals in the study population (approximately 90% of individuals that occupied nest-boxes at the site plus transient and non-resident individuals) were fitted with a leg ring containing a Passive-Integrated Transponder (PIT) tag. The study used data from 139 free-flying, PIT-tagged individuals that interacted with the task. Among these individuals, we recorded and analysed a total of 3117 social association events across 751 distinct dyads. Of these, 648 events involved interactions between affiliates (24 individuals across 18 dyads). The remaining 2469 events involved interactions between unaffiliated individuals (139 individuals across 733 dyads). As we did not interfere with group composition and individuals were free to interact with the task, the research sample is intended to be representative of any wild, freely-interacting jackdaw population.

Sampling strategy

The task was active during the extent of a breeding season (April - July). Sampling commenced prior to the period of peak social foraging activity (post-fledging period, June - July) to maximize recruitment. Early-season sampling was essential to ensure adequate habituation to the task apparatus (to overcome neophobia). The number of birds that visited the task on a given day was stochastic. To maximize sample size, all birds that had been ringed prior to the commencement of the experiment (n=1999) were assigned a treatment class and so were able to participate in the experiment. Sample sizes for affiliate-only analyses were comparable to previous REM studies on jackdaws (Tranmer et al., 2014), whereas sample sizes for non-affiliate analyses and those including all individuals greatly exceeded this and approached sample sizes found in sociological analyses used to demonstrate typical REM applications (see Butts & Marcum, 2017).

- Butts, C.T. & Marcum, C.S. A relational event approach to modelling behavioral dynamics. In: Pilny, A. & Poole, M. (Eds) Group Processes. Computational Social Sciences. Cham: Springer (2017).
- Tranmer, M., Marcum, C.S., Morton, F.B., Croft, D.P. & de Kort, S.R. Using the relational event model (REM) to investigate the

temporal dynamics of animal social networks. Anim. Behav., 101: 99-105 (2015).

Data collection	Data collection was conducted using pairs of automated feeders with Radio-Frequency Identification (RFID) data loggers connected to 'Darwin Board' microcomputers. The data-loggers and microcomputer recorded each bird's arrival and departure from feeder perches along with their unique identifier code. Time of arrival and departure was rounded to the nearest second, but changes in participant ID were detected at a resolution of 250ms. In addition, the 'Darwin Board' logged changes in task states (e.g. opening/closing of doors) occurring in response to coordination events. Task setup, re-stocking of task rewards, maintenance of apparatus and data downloads were performed by JA. In addition, JA collected video recordings of task use for the purposes of validation of data quality.
Timing and spatial scale	Data collection commenced on 24/04/2019 and ended on 02/08/2019. The experiment was run during the breeding season as motivation to engage with novel tasks that provide food rewards is maximal during this period. Data collection was initiated prior to the egg-laying period to promote habituation of both males and females to the task apparatus and ceased once the breeding season ended. The two tasks were placed in the vicinity of nest-boxes in different regions of fields that are home to a breeding colony in Stithians Village, Cornwall, UK.
Data exclusions	Records that featured the RFID codes of tags used by the experimenter to test the functionality of the task at the beginning of each sampling period (i.e. each morning) were removed. Events that contained individuals that did have a PIT-tag, but did not yet have a treatment class assigned (and so were unable to affect task state) at the time the event was recorded were filtered out of the dataset. This scenario occurred because some individuals were fitted with RFID tags during the course of the experiment (e.g. new fledglings or adults that had lost their RFID tag). For these individuals there was a lag (< 24 hours) between the time at which the tag was fitted and the time at which the text files containing treatment class information used by the task apparatus to inform changes in task state were updated. In addition, two individuals and the events in which they participated (37 events: 19 unsuccessful association events, 18 successful) were removed from the dataset as they had duplicate RFID tag codes.
Reproducibility	The experiment featured a wild population of free-flying jackdaws and all ringed members of the population could participate in the experiment. The data collected therefore represents the response of a natural, unaltered social group to an experimental manipulation of social value in a foraging context. To ensure rigour and reproducibility, our analyses feature large sample sizes and use Relational Event Models which are particularly well suited to studying fine-scale changes in social behaviour over time as they do not require any aggregation of data prior to analysis. As an additional robustness check, JA independently ran the analyses conducted by MK, obtaining the same results as those reported in the manuscript. We also ensured reproducibility of our findings by making the data and analysis scripts openly available, allowing analyses to be replicated.
Randomization	Randomization was used to determine treatment class designations. A supervised randomization procedure was used to ensure that an approximately equal number of individuals were assigned to each class and that the distribution of same- versus different-class pairings was approximately equal for key pairings (e.g. known affiliates) upon commencement of the experiment. Balanced assignment of key pairings was deemed necessary as frequency of engagement with the task could not be controlled. Individuals that had been fitted with an RFID-tag prior to the study were assigned a treatment class prior to commencement of the experiment, but individuals that were tagged during the period of the experiment (e.g., newly-fledged juveniles) were assigned a treatment class at random on the day of ringing.
Blinding	Blinding was not necessary for this study. The experiment and data collection were fully automated through RFID data loggers, so there was no scope for experimenter bias.

Did the study involve field work? ☒ Yes ☐ No

Field work, collection and transport

Field conditions	Fieldwork was conducted close to known nest-box breeding colonies near the village of Stithians in Cornwall, UK. Data was collected throughout a breeding season (April - July 2019) on weekdays between approximately 06:00 and 10:00. Weather conditions were not relevant to the research questions under investigation as relevant variables were determined by the structure of the experimental task. Data collection was conducted under most weather conditions, except days with heavy rainfall so as to minimise risk of damage to the electronics of the task apparatus.
Location	Field in the vicinity of Stithians Village, West Cornwall, UK (N 50°11’25.98’’, W 5°10’49.00’’).
Access & import/export	Local landowners kindly granted us permission to work on their land.
Disturbance	To minimize disturbance, the experiment was only run in the mornings. In addition, during this time the researcher (JA) only visited the task apparatus for a brief period at hourly intervals to ensure that the apparatus was not damaged and to replenish food rewards.

Reporting for specific materials, systems and methods

We require information from authors about some types of materials, experimental systems and methods used in many studies. Here, indicate whether each material, system or method listed is relevant to your study. If you are not sure if a list item applies to your research, read the appropriate section before selecting a response.

Materials & experimental systems

n/a	Involved in the study
☒	☐ Antibodies
☒	☐ Eukaryotic cell lines
☒	☐ Palaeontology and archaeology
☐	☒ Animals and other organisms
☒	☐ Clinical data
☒	☐ Dual use research of concern
☒	☐ Plants

Methods

n/a	Involved in the study
☒	☐ ChIP-seq
☒	☐ Flow cytometry
☒	☐ MRI-based neuroimaging

Animals and other research organisms

Policy information about studies involving animals; ARRIVE guidelines recommended for reporting animal research, and Sex and Gender in Research

Laboratory animals	No laboratory animals were used in the study.
Wild animals	We studied free-flying jackdaws (Corvus monedula). Social foraging groups contained adult mated pairs, unpaired adults and juveniles. Ringing of adults and juveniles took place during the experimental period in accordance with established protocols (see 'Ethics oversight').
Reporting on sex	Sex was considered implicitly in study design, as breeding pairs were assigned to treatment classes in such a way as to balance the number of same-class and different-class pairings. However, the overall number of males and females in each treatment class was not identical, as other individuals (i.e., not belonging to nest-box owning pairs) were assigned (at random) to treatment classes in a fashion that did not force an equal balance of sexes. Sex was determined from blood samples via DNA fingerprinting.
Field-collected samples	Blood samples were collected during ringing for use in determination of sex. Wild jackdaws were captured in nest-boxes or ladder traps and colour-ringed by qualified ringers licensed by the British Trust for Ornithology. Whilst in the hand, a small blood sample (<100µl) was taken by applying a needle prick to a superficial blood vessel with a sterile needle and collecting blood in a capillary tube before releasing the bird again. Samples were collected under a U.K. Home Office license (project license 30/3261) and used for molecular sexing to confirm the sex of each individual (Griffiths et al. 1998 Mol. Ecol. 7: 1071-1075). Birds were not brought into captivity and so there are no details about housing, maintenance etc. Blood samples were stored in a -80C freezer until processing.
Ethics oversight	All field protocols were approved by the Biosciences Ethics Panel of the University of Exeter ((2014/577; eCORN000406)) and adhered to the Association for the Study of Animal Behaviour Guidelines for the Treatment of Animals in Behavioural Research and Teaching. Ringing and blood sampling protocols were covered by Home Office (PPL 80/2371) and BTO (C6079, C5752, C5746) licenses.

Note that full information on the approval of the study protocol must also be provided in the manuscript.
